# Clinical features of parainfluenza infections among young children hospitalized for acute respiratory illness in Amman, Jordan

**DOI:** 10.1186/s12879-021-06001-1

**Published:** 2021-04-07

**Authors:** Leigh M. Howard, Danielle A. Rankin, Andrew J. Spieker, Wenying Gu, Zaid Haddadin, Varvara Probst, Herdi Rahman, Rendie McHenry, Claudia Guevara Pulido, John V. Williams, Samir Faouri, Asem Shehabi, Najwa Khuri-Bulos, Natasha B. Halasa

**Affiliations:** 1 Department of Pediatrics, Vanderbilt University School of Medicine, 1161 21st Avenue South, Nashville, TN 37232 USA; 2 Vanderbilt Epidemiology PhD Program, Vanderbilt University School of Medicine, Nashville, TN USA; 3 Department of Biostatistics, Vanderbilt University, Nashville, TN USA; 4 Department of Pediatrics, University of Pittsburgh School of Medicine, UPMC Children’s Hospital of Pittsburgh, Pittsburgh, PA USA; 5 Department of Pediatrics, Al Bashir Hospital, Amman, Jordan; 6 Department of Pathology and Microbiology, University of Jordan, Amman, Jordan; 7 Department of Pediatrics, University of Jordan, Amman, Jordan

**Keywords:** Parainfluenza virus, Acute respiratory illness, Viral pneumonia, Young children, Jordan

## Abstract

**Background:**

Parainfluenza virus (PIV) is a leading cause of acute respiratory illness (ARI) in children. However, few studies have characterized the clinical features and outcomes associated with PIV infections among young children in the Middle East.

**Methods:**

We conducted hospital-based surveillance for ARI among children < 2 years of age in a large referral hospital in Amman, Jordan. We systematically collected clinical data and respiratory specimens for pathogen detection using reverse transcription polymerase chain reaction. We compared clinical features of PIV-associated ARI among individual serotypes 1, 2, 3, and 4 and among PIV infections compared with other viral ARI and ARI with no virus detected. We also compared the odds of supplemental oxygen use using logistic regression.

**Results:**

PIV was detected in 221/3168 (7.0%) children hospitalized with ARI. PIV-3 was the most commonly detected serotype (125/221; 57%). Individual clinical features of PIV infections varied little by individual serotype, although admission diagnosis of ‘croup’ was only associated with PIV-1 and PIV-2. Children with PIV-associated ARI had lower frequency of cough (71% vs 83%; *p* < 0.001) and wheezing (53% vs 60% p < 0.001) than children with ARI associated with other viruses. We did not find a significant difference in supplemental oxygen use between children with PIV-associated infections (adjusted odds ratio [aOR] 1.12, 95% CI 0.66–1.89, *p* = 0.68) and infections in which no virus was detected.

**Conclusions:**

PIV is frequently associated with ARI requiring hospitalization in young Jordanian children. Substantial overlap in clinical features may preclude distinguishing PIV infections from other viral infections at presentation.

**Supplementary Information:**

The online version contains supplementary material available at 10.1186/s12879-021-06001-1.

## Background

Acute respiratory illnesses (ARI) are a leading cause of morbidity and mortality among children globally, and respiratory viruses are increasingly recognized as important etiologies of severe ARI in young children [1, 2]. Parainfluenza virus (PIV) is commonly detected in ARI across all age groups, with highest prevalence typically occurring among young children [2–6]. Clinical features associated with PIV infections range from asymptomatic infection or mild upper respiratory disease to lower respiratory tract illness, with symptoms typically more severe among infants and young children than older children and adults [7–9].

Four distinct serotypes of PIV have been associated with human disease, and individual serotypes may vary with regards to certain clinical features. PIV-3 is the most prevalent serotype and is often associated with lower respiratory disease, including bronchiolitis and pneumonia, while PIV-1 and PIV-2 infections in children are often associated with laryngotracheobronchitis (“croup”) [10, 11]. Due to difficulties in isolation and typical association with milder disease, PIV-4 has often been excluded from many epidemiologic surveillance studies [12], although with advancements in molecular diagnosis, there is increasing evidence that PIV-4 may be associated with more severe, lower respiratory tract disease [2]. Individual PIV serotypes may also vary in terms of their seasonal patterns of detection. In temperate regions, peak PIV-2 and PIV-3 detections typically occur annually in the late spring (April–June) and autumn (October–November), while PIV-1 exhibits biennial peaks later in the year in odd years [11, 13], although serotype-specific seasonality information is scarce in many regions [14].

Despite the importance of PIV as a common etiology of severe respiratory illnesses in children, many studies of PIV infections in children have focused on those with pneumonia and have excluded children with other forms of ARI [12, 15]. We conducted a prospective study of ARI-associated hospitalizations among young children < 2 years of age over a three-year period in Amman, Jordan. We sought to define the clinical features of PIV infections in young children and to compare these among serotypes 1, 2, 3, and 4 as well as with ARI associated with other respiratory viruses.

## Methods

### Study design and setting

We enrolled children less than 2 years of age admitted with ARI (fever and/or respiratory symptoms) to Al-Bashir Government Hospital in Amman, Jordan from March 2010 to March 2013. Detailed characterization of the study population, setting, and design have been previously published [16, 17]. Briefly, children were eligible if they were admitted with fever and/or a respiratory symptom (e.g. cough, shortness of breath, wheezing) and one of the following admission diagnoses: ARI, apnea, asthma exacerbation, bronchiolitis, bronchopneumonia, croup, cystic fibrosis exacerbation, febrile seizure, fever without localizing signs, respiratory distress, pneumonia, pneumonitis, pertussis, pertussis-like cough, rule-out sepsis (evaluation of neonates with fever), upper respiratory infection (URI) and/or a lower respiratory tract illness. Lower respiratory tract illness (LRTI) was defined as an admission diagnosis of asthma, bronchiolitis, bronchopneumonia, pneumonia, respiratory distress, or wheezing and/or the presence of retractions/accessory muscle use or wheezing on examination [16]. Children with neutropenia secondary to chemotherapy and newborns who had never been discharged from the hospital after birth were excluded from the study. Active, year-round, hospital-based surveillance was conducted to recruit eligible children during 5 days of each week (Sunday-Thursday) within 48 h of hospital admission.

Al-Bashir Hospital is the major government-run referral center that serves Amman (estimated population > 2 million). The hospital provided care for an estimated 50–60% of children residing in Amman during the study period [16]. During the study period, 11,230 of the 17,557 children (64%) admitted to the pediatric wards were under 2 years of age. Written informed consent was obtained from parents or guardians of children prior to enrollment and initiation of study activities. The institutional review boards of the University of Jordan, the Jordanian Ministry of Health, and Vanderbilt University approved the study. All methods were performed in accordance with relevant guidelines and regulations.

### Data collection

After informed consent, trained local research staff interviewed parents/legal guardians in Arabic using a standardized questionnaire that included Arabic and English translations to record demographic, clinical, and socioeconomic data, including the presence of underlying medical conditions (UMCs) and household smoke (cigarette and/or hookah pipe) exposure. After hospital discharge, charts were abstracted for the following: antibiotic use, blood culture results, chest radiography results, oxygen use, intensive care unit (ICU) admission, mechanical ventilation (MV), length of stay (LOS) in the hospital, and status at discharge. All data were entered into a standardized, secure Research Electronic Data Capture (REDCap) database (Vanderbilt University, Nashville, Tennessee, USA). We performed data quality checks on a minimum of 10% of the charts and verified data from all case report forms after entry. The following were considered UMCs: diabetes, heart disease, Down syndrome, kidney disease, sickle cell disease, cystic fibrosis, cancer, genetic/metabolic, cerebral palsy, neurological, mental retardation/developmental delay, seizure disorder, chronic diarrhea (> 2 weeks), gastroesophageal reflux disease, immunodeficiency, asthma/reactive airway disease, or liver disease.

### Sample collection and testing

Nasal and throat swabs were collected from enrolled subjects and combined in transport medium (M4RT, Remel, USA), aliquoted into MagMAX Lysis/Binding Solution Concentrate (Life Technologies, USA), snap-frozen and then stored at − 80 °C. Aliquots were shipped on dry ice to Nashville, Tennessee, USA, and were tested by individual single-plex RT-PCR for 16 viruses: respiratory syncytial virus (RSV); human rhinovirus (HRV); human metapneumovirus (HMPV); influenza (influenza) A, B and C; parainfluenza virus (PIV) 1, 2, 3, and 4; human adenovirus (AdV), human coronaviruses (HCoV) HKU1, OC43, 229E, and NL63, and Middle East respiratory syndrome coronavirus (MERS-CoV) [18].

### Statistical analysis

We compared common clinical features and seasonality of detection in patients with PIV-1, PIV-2, PIV-3 and PIV-4-associated respiratory illness, as defined by detection of each PIV serotype from a respiratory sample by PCR, including co-detections of other respiratory viruses. Next, we compared features of PIV-only respiratory illness, excluding co-detections with other viruses, to ARI associated with detection of other viruses (sole or co-detection of hMPV, RSV, AdV, MERS-CoV, influenza, or HRV), and ARI with no virus detected. These categories were mutually exclusive. Chi-square and two sample t-tests allowing unequal variances were used to compare categorical and continuous variables, respectively, between groups.

Linear regression with robust standard errors was used to evaluate the association between ARI etiologic category (PIV, other viruses, no virus) and time to hospital discharge (hospital length of stay [LOS] measured in hours). Model covariates included age, sex, breastfeeding status, antibiotic use prior to admission, duration of symptoms prior to admission, smoke exposure, and presence of at least one UMC. Logistic regression with robust standard errors was used to evaluate the association between ARI etiologic category (PIV, other viruses, no virus) and the need for supplemental oxygen as a surrogate indicator of illness severity, using the same covariates as in the linear regression model. All analyses were conducted using statistical software StataIC 16.0 (StatCorp LLC, College Station, TX). For all statistical tests, we used a nominal significance level of α = 0.05.

## Results

### PIV-associated acute respiratory illness

**Fig. 1:**
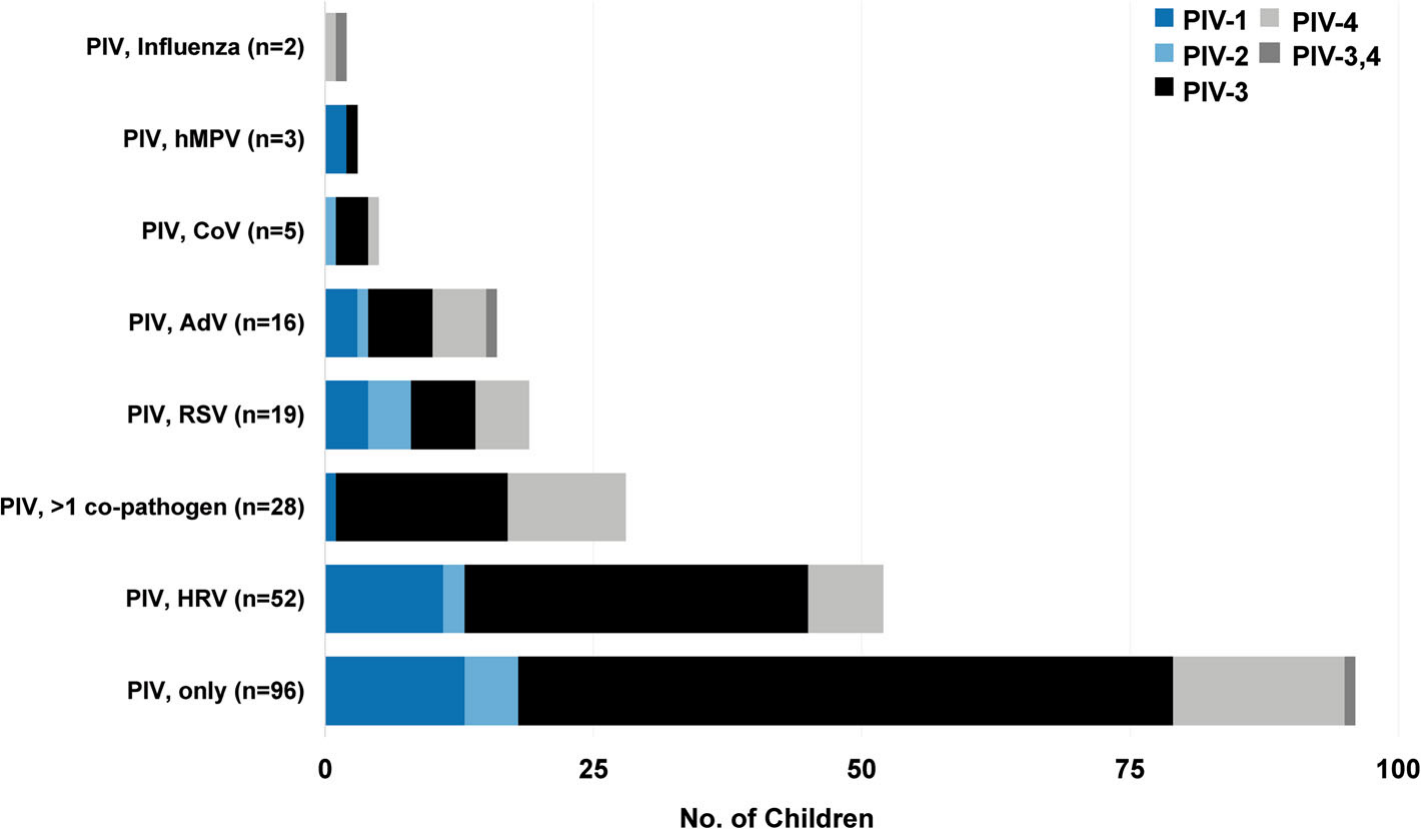
Co-detection of parainfluenza viruses with other respiratory viruses

Among 3168 children admitted with ARI and included in this study, PIV was detected by nasal/throat swab PCR in 221 (7.0%; Supplementary Figure 1). Other viruses were commonly co-detected with PIV (125/221, 57%; Fig. 1); most commonly HRV (24%), RSV (9%), and adenovirus (7%). PIV-3 was the most commonly detected of the PIV serotypes (128/221, 58%, Table 1), followed by PIV-4 (49/221; 22%), PIV-1 (34/221; 15%), and PIV-2 (13/221; 6%). PIV-3 and PIV-4 were codetected in 3 children. Three of 95 children with PIV infection from whom a blood culture was collected had a positive blood culture result [*Staphylococcus aureus* (22 day old with discharge diagnosis of sepsis; *n* = 1), *Candida spp.* (40 day old with discharge diagnoses of pneumonia and sepsis; *n* = 1), *Micrococcus spp*. (suspected contaminant; *n* = 1)].

**Table 1:** Characteristics of hospitalized children with PIV-1, PIV-2, PIV-3, and PIV-4 including co-detections with other pathogens

	PIV-1 (***n*** = 34) No. (%)	PIV-2 (***n*** = 13) No. (%)	PIV-3^**a**^ (***n*** = 125) No. (%)	PIV-4^**a**^ (***n*** = 46) No. (%)	***p***-value
Female sex	16 (47.1)	6 (46.2)	48 (38.4)	21 (45.7)	0.714
Median age, months (IQR)	9.0 (3.5–16.1)	5.4 (2.6–8.6)	3.2 (1.7–8.9)	2.6 (1.7–9.1)	**0.003**
Age group					
< 6 months	13 (38.2)	7 (53.9)	80 (64.0)	29 (63.0)	**0.028**
6 to < 12 months	7 (20.6)	5 (38.5)	23 (18.4)	8 (17.4)	
12 to < 24 months	14 (41.2)	1 (7.7)	22 (17.6)	9 (19.6)	
Received influenza vaccine	0 (0)	0 (0)	1 (1.0)	0 (0)	0.861
Breastfed, any	30 (88.2)	13 (100)	92 (73.6)	37 (80.4)	0.059
Any underlying medical condition^b^	3 (8.8)	2 (15.4)	19 (15.2)	6 (13.0)	0.810
Household smoke exposure	23 (67.7)	10 (76.9)	95 (76.0)	37 (80.4)	0.620
Symptom duration prior to admission, days (IQR)	3.5 (2–5)	2 (2–4)	3 (2–5)	2 (1–5)	0.253
Antibiotics prior to admission	17 (50.0)	3 (23.1)	42 (33.6)	19 (41.3)	0.210
Antibiotics during hospitalization	33 (97.1)	9 (69.2)	115 (92.0)	44 (95.7)	**0.010**
Clinical presentation					
Fever/feverish^c^	21 (61.8)	9 (69.2)	77 (61.6)	27 (58.7)	0.923
Cough^c^	30 (88.2)	10 (76.9)	90 (72.0)	36 (78.3)	0.258
Shortness of breath^c^	23 (67.7)	9 (69.2)	55 (44.0)	28 (60.9)	**0.045**
Flaring/Retractions^d^	14 (41.2)	5 (38.5)	36 (28.8)	15 (32.6)	0.541
Retractions, only^d^	2 (5.9)	1 (7.7)	4 (3.2)	4 (8.7)	0.492
Wheezing^d^	20 (58.8)	6 (46.2)	62 (49.6)	22 (47.8)	0.750
Admission diagnosis					
Reactive airway disease	3 (8.8)	1 (7.7)	4 (3.2)	1 (2.2)	0.387
Bronchiolitis	6 (17.7)	3 (23.1)	14 (11.2)	10 (21.7)	0.275
Bronchopneumonia	14 (41.2)	4 (30.8)	48 (38.4)	13 (28.3)	0.563
Croup	2 (5.9)	1 (7.7)	0 (0.0)	0 (0.0)	**0.010**
Febrile seizure	2 (5.9)	1 (7.7)	4 (3.2)	1 (2.2)	0.697
Pneumonia	4 (11.8)	0 (0.0)	12 (9.6)	5 (10.9)	0.649
Pertussis-like cough	0 (0.0)	0 (0.0)	9 (7.2)	4 (8.7)	0.269
Rule-out sepsis	5 (14.7)	3 (23.1)	40 (32.0)	11 (23.9)	0.211
Classified as LRTI	28 (82.4)	9 (69.2)	84 (67.2)	31 (67.4)	0.382
Chest radiograph					
Abnormal	23/31 (74.2)	8/11 (72.7)	78/116 (67.2)	28/44 (63.4)	0.785
Required supplemental oxygen	11 (32.4)	2 (15.4)	35 (28.0)	14 (30.4)	0.695
Required ICU admission	5 (14.7)	0 (0.0)	9 (7.2)	3 (6.5)	0.318
Required mechanical ventilation	2 (5.9)	0 (0.0)	5 (4.0)	0 (0.0)	0.393
Length of stay (days), median (IQR)	3 (2–6)	5 (3–8)	6 (3–8)	5 (3–8)	0.161
Codetected with ≥1 other virus	21 (61.8)	8 (61.5)	64 (51.2)	30 (65.2)	0.338
Death	0 (0.0)	0 (0.0)	0 (0.0)	1 (2.2)	0.431

^a^ Individual serotypes detected and distinguished by PCR; excludes 3 cases of codetection of PIV-3 and PIV-4

^+^ *p* < 0.05 considered statistically significant, indicated by bold text

^b^ UMC defined as at least one of the following: diabetes, heart disease, Down syndrome, kidney disease, sickle cell disease, cystic fibrosis, cancer, genetic/metabolic, cerebral palsy, neurological, mental retardation/developmental delay, seizure disorder, chronic diarrhea (eg, > 2 weeks), gastroesophageal reflux disease, immunodeficiency, asthma/reactive airway disease, liver disease

^c^ Reported by parent/legal guardian

^d^ Collected through clinical exam

### Seasonality of PIV detections

**Fig. 2:**
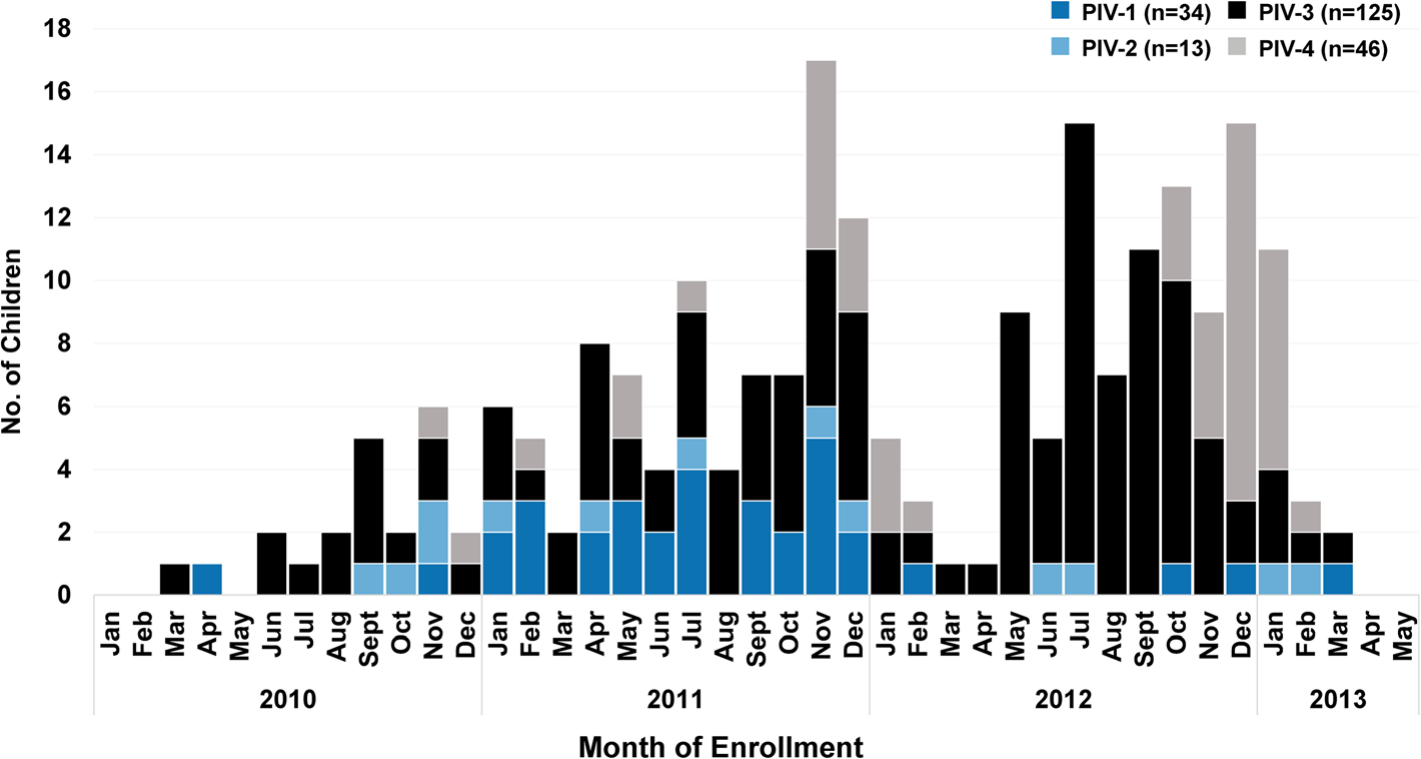
Detection of parainfluenza virus by month and year among Jordanian children admitted with acute respiratory illness from March 2010 to March 2013

While PIV detections occurred throughout the year during our study, peak PIV detections in 2011 occurred in November–December (Fig. 2), while in 2012 a earlier peak occurred in July in addition to the winter (November 2012 to January 2013) peak. PIV detections in the spring-summer months were primarily contributed by PIV-3. The majority of PIV-1 detections occurred in 2011.

### Clinical features of PIV-1, PIV-2, PIV-3, and PIV-4 infections

The clinical features of infections associated with individual PIV serotypes, inclusive of co-detections with other viruses, are displayed in Table 1. The median age of hospitalized children with PIV-3 and PIV-4 detections was significantly lower than those with PIV-1 or PIV-2 (Table 1). The proportion of children with an underlying medical condition was similar among children with infection associated with each of the PIV serotypes. Greater than 90% of children with PIV-1, PIV-3, and PIV-4 infections received antibiotics during hospitalization, compared to only 69% of those hospitalized with PIV-2 associated infections (Table 1). The clinical symptoms of PIV infections did not vary significantly according to individual serotype. Bronchopneumonia, rule-out sepsis, and bronchiolitis were the most common admission diagnoses assigned to PIV 1–4 infections; an admission diagnosis of “croup” was only assigned to admissions associated with PIV-1 or PIV-2, while “pertussis-like cough” was assigned only to PIV-3 and PIV-4 associated admissions. Only 2/13 (15%) children with PIV-2-associated infections required supplemental oxygen, compared to 32, 28, and 30% of children with PIV-1, PIV-3, and PIV-4-associated infections, respectively, however this difference was not statistically significant (*p* = 0.695). The clinical features of infections associated with individual PIV serotypes, excluding co-detections with other viruses, are displayed in Supplementary Table 1.

### Features of PIV-associated infections compared to other viral infections

After exclusion of infections in which PIV was codetected with other respiratory viruses, the clinical features of children with PIV-only associated ARI compared to other respiratory viruses or no virus detection are displayed in Table 2. Compared to both groups, PIV-only children had higher mean age (Table 2). Children with PIV had a higher frequency of fever (63%) than those with ARI associated with other viruses (53%; *p* < 0.001), but they had a lower frequency of nasal flaring/chest retractions (33% vs. 46%, p < 0.001) and wheezing (53% vs. 60%, p < 0.001). A higher frequency of children with PIV-only and other virus-associated ARI met the LRTI definition, had longer symptom onset prior to admission, and had higher occurrence of cough, nasal flaring/chest retractions, and wheezing compared to children with no virus detected. The most common admission diagnoses assigned to PIV-only group in descending order were bronchopneumonia, rule-out sepsis, pneumonia, and bronchiolitis while bronchopneumonia, bronchiolitis, rule-out sepsis, pneumonia were the most common admission diagnoses for any PIV detection (Tables 1 and 2). The frequency of croup as an admission diagnosis was higher in children with PIV-only compared to the other groups, while the frequencies of bronchiolitis, bronchopneumonia, and pneumonia were higher among the PIV-only and other virus group compared to the children in whom no virus was detected. In unadjusted comparisons, fewer children with PIV-associated infection required supplemental oxygen (23%) or ICU admission (6%) than those with ARI associated with other viruses (35 and 9%, respectively; *p* < 0.001 and *p* = 0.048).

**Table 2:** Characteristics of hospitalized children with PIV-associated respiratory illness compared to other-viral and virus-negative respiratory illness

	PIV only* (***n*** = 96) No. (%)	Other virus, not PIV (***n*** = 2413) No. (%)	No virus (***n*** = 534) No. (%)	***p***-value^**+**^
Female sex	39 (40.6)	954 (39.5)	209 (39.1)	0.960
Median age, months (IQR)	4.2 (1.7–10.8)	3.8 (1.8–8.5)	2.3 (1.1–7.6)	**< 0.001**
Age group				
< 6 months	54 (56.3)	1544 (64.0)	382 (71.5)	**0.001**
6 to < 12 months	19 (19.8)	499 (20.7)	79 (14.8)	
12 to < 24 months	23 (24.0)	370 (15.3)	73 (13.7)	
Received influenza vaccine	0 (0.0)	5 (0.21)	1 (0.2)	0.264
Breastfed	78 (81.3)	2034 (84.3)	453 (84.8)	0.674
Any underlying medical condition^a^	12 (12.5)	269 (11.2)	75 (14.0)	0.164
Asthma/reactive airway disease	1 (1.0)	65 (2.7)	10 (1.9)	0.285
Household smoke exposure	74 (77.1)	1851 (76.7)	406 (76.0)	0.939
Symptom duration prior to admission	3 (1.5–4)	3 (2–5)	2 (1–3)	**0.002**
Antibiotics during hospitalization	89 (92.7)	2193 (90.9)	489 (91.6)	0.746
Clinical presentation				
Fever/feverish^b^	60 (62.5)	1279 (53.0)	347 (65.0)	**< 0.001**
Cough^b^	68 (70.8)	1990 (82.5)	207 (38.8)	**< 0.001**
Shortness of breath^b^	48 (50.0)	1543 (64.0)	172 (32.2)	**< 0.001**
Flaring/Retractions^c^	32 (33.3)	1099 (45.5)	121 (22.7)	**< 0.001**
Retractions, only^c^	5 (5.2)	240 (10.0)	30 (5.6)	**0.003**
Wheezing^c^	51 (53.1)	1455 (60.3)	189 (35.4)	**< 0.001**
Admission diagnosis				
Reactive airway disease	4 (4.2)	123 (5.1)	14 (2.6)	**0.047**
Bronchiolitis	10 (10.4)	483 (20.0)	31 (5.8)	**< 0.001**
Bronchopneumonia	34 (35.4)	818 (33.9)	121 (22.7)	**< 0.001**
Croup	2 (2.1)	6 (0.3)	1 (0.2)	**0.005**
Febrile seizure	5 (5.2)	47 (2.0)	28 (5.2)	**< 0.001**
Pneumonia	14 (14.6)	338 (14.0)	34 (6.4)	**< 0.001**
Pneumonitis	0 (0.0)	1 (0.04)	1 (0.19)	0.477
Pertussis-like cough	6 (6.3)	182 (7.5)	22 (4.1)	**0.018**
Rule-out sepsis	27 (28.1)	559 (23.2)	282 (52.8)	**< 0.001**
Classified as LRTI	68 (70.8)	1878 (77.8)	230 (43.1)	**< 0.001**
Chest radiograph				
Abnormal	60/90 (66.7)	1734/2302 (75.3)	203/454 (44.7)	**< 0.001**
Required supplemental oxygen	22 (22.9)	829/2384 (34.8)	122/532 (22.9)	**< 0.001**
Required ICU admission	6 (6.3)	205 (8.5)	62 (11.6)	**0.048**
Required mechanical ventilation	3 (3.1)	82/2383 (3.4)	22/532 (4.1)	0.717
Length of stay (days), median (IQR)	5 (3–8)	5 (3–7)	5 (3–8)	0.154
Death	0 (0.0)	21 (1.0)	9 (2.0)	0.140

^*^ One child with both PIV 3 and PIV 4 serotypes is included in the PIV only group

^+^ *p* < 0.05 considered statistically significant, indicated by bold text

^a^ UMC defined as at least one of the following: diabetes, heart disease, Down syndrome, kidney disease, sickle cell disease, cystic fibrosis, cancer, genetic/metabolic, cerebral palsy, neurological, mental retardation/developmental delay, seizure disorder, chronic diarrhea (eg, > 2 weeks), gastroesophageal reflux disease, immunodeficiency, asthma/reactive airway disease, liver disease

^b^ Reported by parent/legal guardian

^c^ Collected through clinical exam

### Multivariable analysis: hospital length of stay

We used linear regression to compare the mean length of hospital stay between children with PIV-associated and other viral ARI compared to ARI in which no viruses were detected (reference), adjusting for age, sex, breastfeeding status, antibiotic use prior to admission, duration of symptoms prior to admission, smoke exposure, and presence of at least one UMC. Based on this model, we estimated the mean hospital length of stay to be 0.51 days longer in the PIV group compared to those with no viruses detected (95% confidence interval [CI] -0.50, 1.52, *p* = 0.32). Further, we estimated the mean hospital length of stay to be 0.30 days shorter for ARI associated with other viruses compared to ARI in which no viruses were detected (95% CI -0.70, 0.10, *p* = 0.14).

### Multivariable analysis: supplemental oxygen use

**Fig. 3:**
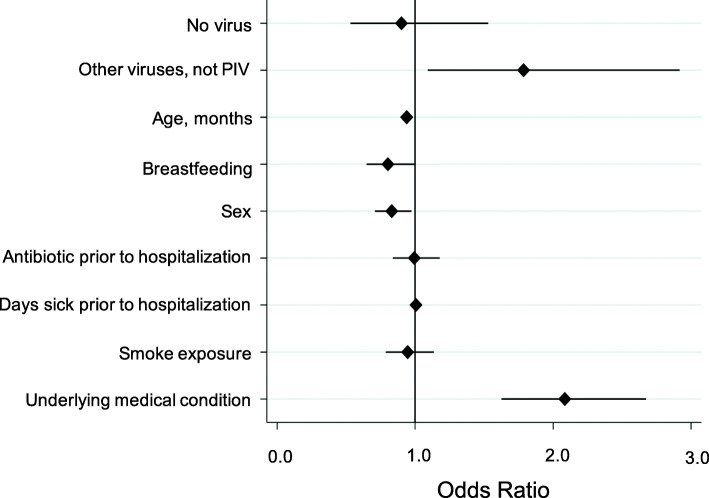
Association between PIV-associated infections, other infections, and supplemental oxygen use among young Jordanian children admitted with acute respiratory illness

We did not find sufficient evidence of a difference in supplemental oxygen use between children with PIV-associated infections (reference) adjusted odds ratio and infections in which no virus was detected (adjusted odds ratio [aOR] 0.90, 95% CI 0.53–1.53, *p* = 0.70; Fig. 3). Infections with viruses other than PIV and the presence of an underlying medical condition were associated with significantly increased odds of supplemental oxygen use (aOR 1.79, 95% CI 1.09–2.92, *p* = 0.02 and aOR 2.08, 95% CI 1.62–2.67, *p* < 0.001, respectively).

## Discussion

While RSV [16] and HMPV [19] have previously been recognized as important etiologies of ARI associated with hospitalization in young Jordanian children, our findings underscore the importance of PIV as an etiology of ARI and LRTI requiring hospitalization in young children in Jordan. We identified a higher frequency of fever and a lower frequency of nasal flaring/retractions and wheezing in children with PIV compared to other ARI in unadjusted analyses, and significantly lower odds of supplemental oxygen use in children with PIV compared to other viral infections. However, there was substantial overlap in the presenting signs and symptoms of PIV infections and those of other viral etiologies, limiting the ability to distinguish these etiologies clinically at the time of presentation. We also found substantial overlap in the clinical features of infections associated with individual PIV serotypes but substantial variability in the seasonality of individual PIV serotype detections. PIV-1 and PIV-2 were the only PIV serotypes associated with an admission diagnosis of croup, consistent with literature that these serotypes are the most common pathogens associated with laryngotrachobronchitis [8]. In addition, our report is among the first to include clinical and epidemiological information regarding PIV-4 infections in young children in the Middle East.

In our study, PIV was detected in 7% of children who presented with both fever and/or respiratory symptoms. Recent reports have focused on the role of PIV in respiratory illnesses in children, but many of these have focused on severe illnesses including pneumonia. For example, in the multicenter Etiology of Pneumonia in the Community (EPIC) study in the United States, PIV infection was detected in 6.6% of cases of radiographically-confirmed pneumonia in hospitalized children, and these infections were associated with similar severity as infections with other respiratory viruses [12]. In the international Pneumonia Etiology for Child Health (PERCH) study, PIV (including PIV-4) was detected in 12% of pneumonia cases and 5.9% of controls, and PIV was among the five most common pathogens in five of the seven study sites, which did not include the Middle East [2, 15]. Our results were consistent with this study, reporting that PIV was the fifth most commonly detected virus after RSV, HRV, AdV, and HMPV [17]. Although our study was limited to a single center, we uniquely included the full spectrum of respiratory conditions associated with PIV, such as laryngotracheobronchitis and bronchiolitis, in a setting in which household smoke exposure, an established risk factor associated with acute respiratory illnesses and respiratory outcomes in children, [20, 21] was relatively high [12].

We observed the majority of PIV-1 detections were in 2011, an odd year, which is consistent with prior reports of seasonal detections of PIV-1 from United States, in which PIV-1 often exhibits biennial peaks [11, 22]. In 2012, the earlier peak was dominated by PIV-3, consistent with other studies that report that PIV-3 primarily circulates annually during the summer months [11, 22]. We did not see predominant circulation of PIV-2 during even years although detection of few cases of PIV-2 in our hospitalized cohort limited seasonality assessment for this serotype, for which many milder infections managed in the outpatient setting may not have been captured. Few studies have reported the seasonal patterns of detection of PIV-4; we primarily observed detections in the late autumn and winter months in our cohort.

Our study has several strengths, including active surveillance and comprehensive respiratory viral testing. The study also has several limitations. Lower respiratory tract specimens were infrequently available. Healthy controls were not included. Although PIV is thought to be infrequently detected in asymptomatic young children [23], it has been detected more frequently among healthy controls in other studies [2]. Frequent co-detections of PIV with other respiratory viruses limited our ability to compare sole detections of PIV to other viruses and to compare the clinical features of individual PIV serotypes with robust conclusions.

## Conclusions

In summary, PIV infections were common in children hospitalized with ARI. There were few distinguishing clinical features at presentation, yet several differences in seasonal detection patterns observed, according to PIV serotype. No clinical characteristics were identified that could reliably distinguish PIV from ARI caused by other viruses at presentation, although PIV-associated ARI were less frequently associated with supplemental oxygen use. Our findings suggest that all four PIV serotypes are important etiologies of ARI associated with hospitalization in young Jordanian children. Such surveillance may provide more conclusive characterization of the epidemiology of PIV in the Middle East to support the need for evaluation of future vaccines and antivirals in this region [24, 25].

## Supplementary Information

**Additional file 1.**

## Data Availability

The datasets used and/or analysed during the current study are available from the corresponding author on reasonable request.

## Abbreviations

AdV: Human adenovirus
aOR: Adjusted odds ratio
ARI: Acute respiratory illness
CI: Confidence interval
EPIC: Etiology of Pneumonia in the Community
HCoV: Human coronaviruses
HMPV: Human metapneumovirus
HRV: Human rhinovirus
ICU: Intensive care unit
LOS: Length of stay
LRTI: Lower respiratory tract illness
MERS-CoV: Middle East respiratory syndrome coronavirus
MV: Mechanical ventilation
PERCH: Pneumonia Etiology for Child Health
PIV: Parainfluenza virus
REDCap: Research Electronic Data Capture
RSV: Respiratory syncytial virus
URI: Upper respiratory infection
UMCs: Underlying medical conditions
