# Supplementary material for: Clinical features of parainfluenza infections among young children hospitalized for acute respiratory illness in Amman, Jordan
Source: BMC Infect Dis. 2021 Apr 7;21:323. doi: 10.1186/s12879-021-06001-1 (PMC8024934; doi:10.1186/s12879-021-06001-1)
Supplement: Supplementary file 1 — Additional file 1. [file 12879_2021_6001_MOESM1_ESM.docx]

**Supplementary Appendix**

**Supplementary Figure 1.** Viral testing results among 3168 children less than two years of age hospitalized in Amman, Jordan for acute respiratory illness from March 2010 to March 2013

**
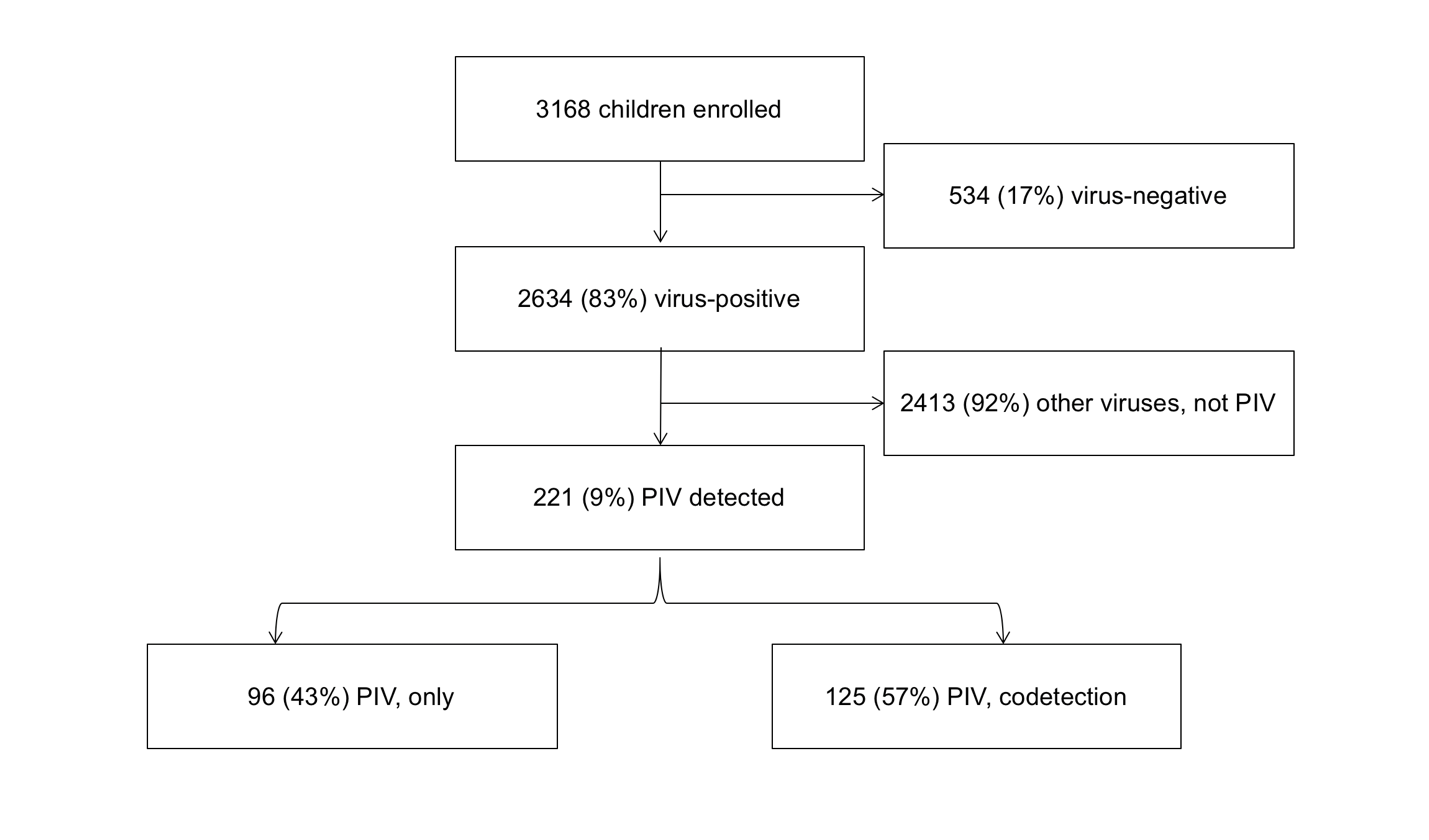
**

| **Supplemental Table 1**. Characteristics of hospitalized children with PIV-1, PIV-2, PIV-3, and PIV-4 excluding co-detections with other pathogens | | | | | |
| --- | --- | --- | --- | --- | --- |
|  | **PIV-1**  **(n=13)**  **No. (%)** | **PIV-2**  **(n=5)**  **No. (%)** | **PIV-3***  **(n=61)**  **No. (%)** | **PIV-4***  **(n=16)**  **No. (%)** | p-value |
| Female sex | 6 (46.2) | 2 (40.0) | 21 (34.4) | 9 (56.3) | 0.377 |
| Median age, months (IQR) | 10.8 (4.3-16.3) | 8.3 (6.6-9.8) | 3.4 (1.5-9.7) | 2.2 (1.5-7.2) | **0.006** |
| Age group |  |  |  |  |  |
| <6 months | 5 (38.5) | 1 (20.0) | 37 (60.7) | 11 (68.8) | **0.006** |
| 6 to <12 months | 2 (15.4) | 4 (80.0) | 9 (14.8) | 3 (18.8) |  |
| 12 to <24 months | 6 (46.2) | 0 (0) | 15 (24.6) | 2 (12.5) |  |
| Received influenza vaccine | 0 (0.0) | 0 (0.0) | 0 (0.0) | 0 (0.0) | 0.964 |
| Breastfed, any | 11 (84.6) | 5 (100) | 49 (80.3) | 13 (81.3) | 0.230 |
| Any underlying medical condition^#^ | 1 (7.7) | 2 (40.0) | 8 (13.1) | 0 (0.0) | **0.011** |
| Household smoke exposure | 9 (69.2) | 4 (80.0) | 48 (78.7) | 12 (75.0) | 0.924 |
| Symptom duration prior to admission, days (IQR) | 4.0 (1-6) | 4 (3-5) | 3 (2-4) | 2 (1-3) | 0.074 |
| Antibiotics prior to admission | 8 (61.5) | 2 (40.0) | 20 (32.8) | 6 (37.5) | 0.355 |
| Antibiotics during hospitalization | 13 (100) | 4 (80.0) | 57 (93.4) | 14 (87.5) | 0.560 |
| Clinical presentation |  |  |  |  |  |
| Fever/feverish^a^ | 7 (53.9) | 4 (80.0) | 43 (70.5) | 5 (31.3) | **0.040** |
| Cough^a^ | 10 (76.9) | 4 (80.0) | 40 (65.6) | 13 (81.3) | 0.644 |
| Shortness of breath^a^ | 23 (67.7) | 9 (69.2) | 55 (44.0) | 28 (60.9) | **0.045** |
| Flaring/Retractions^b^ | 5 (38.5) | 2 (40.0) | 18 (29.5) | 7 (43.7) | 0.747 |
| Retractions, only^b^ | 1 (7.7) | 0 (0.0) | 2 (3.3) | 2 (12.5) | 0.614 |
| Wheezing^b^ | 7 (53.9) | 2 (40.0) | 31 (50.8) | 10 (62.5) | 0.749 |
| Admission diagnosis |  |  |  |  |  |
| Reactive airway disease | 2 (15.4) | 1 (20.0) | 1 (1.6) | 0 (0.0) | 0.062 |
| Bronchiolitis | 1 (7.7) | 0 (0.0) | 3 (4.9) | 6 (37.5) | **0.004** |
| Bronchopneumonia | 2 (15.4) | 3 (60.0) | 25 (41.0) | 3 (18.8) | 0.085 |
| Croup | 2 (15.4) | 0 (0.0) | 0 (0.0) | 0 (0.0) | **0.011** |
| Febrile seizure | 1 (7.7) | 1 (7.7) | 4 (3.2) | 1 (2.2) | 0.697 |
| Pneumonia | 4 (30.8) | 0 (0.0) | 8 (13.1) | 2 (12.5) | 0.417 |
| Pertussis-like cough | 0 (0.0) | 0 (0.0) | 4 (6.6) | 2 (12.5) | 0.673 |
| Rule-out sepsis | 2 (15.4) | 0 (0.0) | 23 (37.7) | 2 (12.5) | 0.088 |
| Classified as LRTI | 10 (76.9) | 4 (80.0) | 41 (67.2) | 12 (75.0) | 0.849 |
| Chest radiograph |  |  |  |  |  |
| Abnormal | 9/13 (81.8) | 4/5 (100) | 58/61 (62.1) | 10 (62.5) | 0.365 |
| Required supplemental oxygen | 5 (38.5) | 1 (20.0) | 12 (19.7) | 4 (25.0) | 0.644 |
| Required ICU admission | 3 (23.1) | 0 (0.0) | 2 (3.3) | 1 (6.3) | 0.107 |
| Required mechanical ventilation | 1 (7.7) | 0 (0.0) | 2 (3.3) | 0 (0.0) | 0.807 |
| Length of stay (days), median (IQR) | 3 (2-8) | 5 (3-7) | 6 (3-8) | 4 (2-8) | 0.919 |
| Death | 0 (0.0) | 0 (0.0) | 0 (0.0) | 0 (0.0) | -- |
| * Individual serotypes detected and distinguished by PCR; excludes 1 case of codetection of PIV-3 and PIV-4  ^+^ p<0.05 considered statistically significant, indicated by bold text  ^#^ UMC defined as at least one of the following: diabetes, heart disease, Down syndrome, kidney disease, sickle cell disease, cystic fibrosis, cancer, genetic/metabolic, cerebral palsy, neurological, mental retardation/developmental delay, seizure disorder, chronic diarrhea (eg, >2 weeks), gastroesophageal reflux disease, immunodeficiency, asthma/reactive airway disease, liver disease  ^a^ Reported by parent/legal guardian  ^b^ Collected through clinical exam | | | | | |
